# Supplementary material for: Antitumour Effects of Astaxanthin and Adonixanthin on Glioblastoma
Source: Mar Drugs. 2020 Sep 18;18(9):474. doi: 10.3390/md18090474 (PMC7551886; doi:10.3390/md18090474)
Supplement: Supplementary file 1 [file marinedrugs-18-00474-s001.pdf]

Supplemental table 1. Astaxanthin or adonixanthin levels in the mouse tissues without brain

Tissues	Astaxanthin		Adonixanthin	
	trans	*cis*	*trans*	*cis*
Heart	57.81 ± 11.61	66.71 ± 30.75	134.55 ± 33.00*	6.37 ± 5.71
Lung	64.26 ± 29.75	41.91 ± 36.72	1841.33 ± 1181.79*	103.03 ± 66.09
Spleen	74.16 ± 20.83*	13.62 ± 2.47	255.03 ± 45.17*	22.13 ± 3.47
Kidney	59.90 ± 27.80	24.73 ± 14.34	734.62 ± 111.01*	38.99 ± 6.12
Liver	95.00 ± 22.13*	13.57 ± 6.08	1817.92 ± 738.28*	108.52 ± 42.28

Data are shown as mean ± SEM (n = 4). ng/g. *$p < 0.05$, **$p < 0.01$ vs. *cis*-form group (Mann-Whitney *U*-test).

Supplemental Figure 1

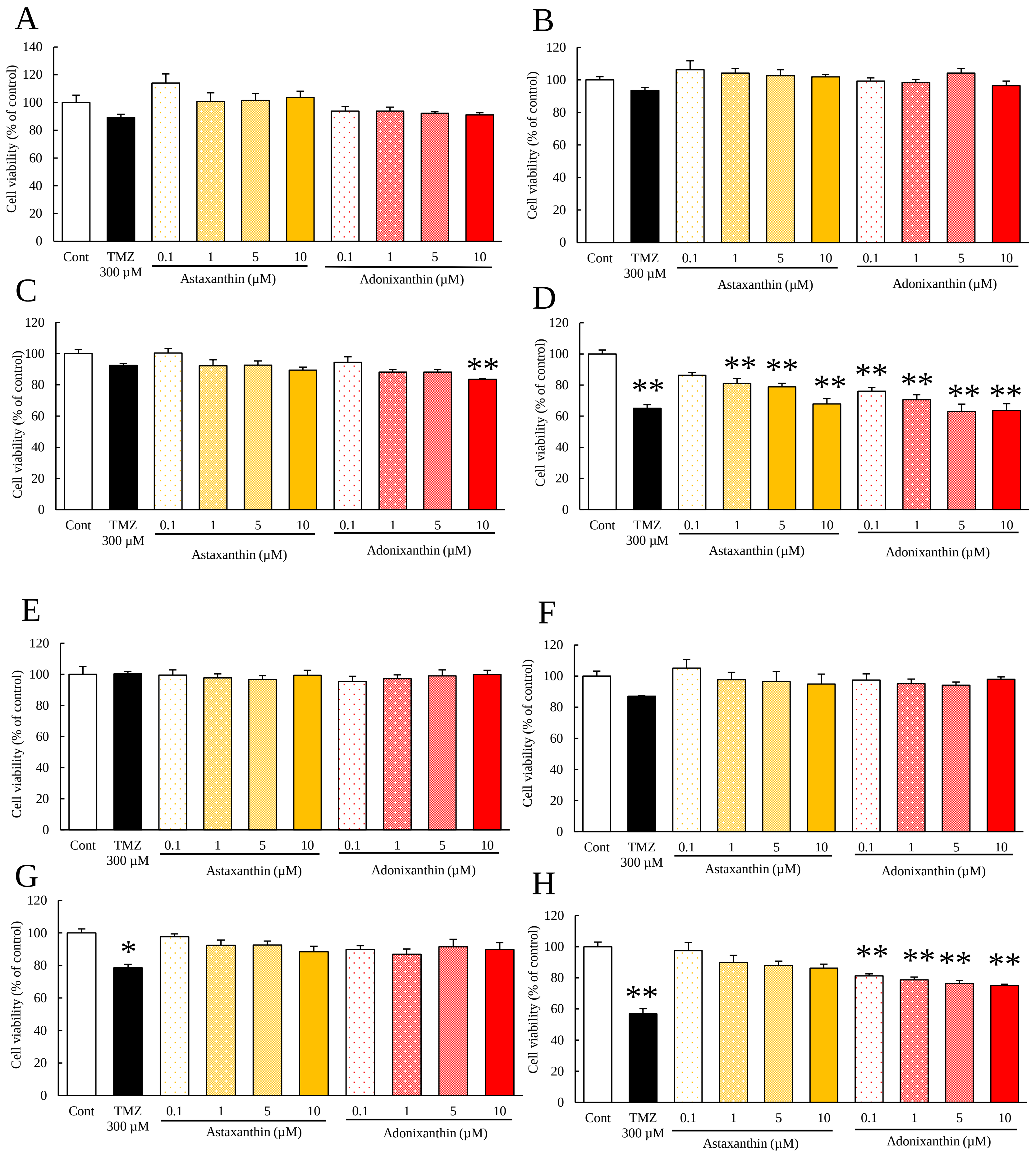

Supplemental figure 1. Cell viability of mouse and human glioblastoma cell line with astaxanthin and adonixanthin.

(A-H) These graphs show the cell viability of GL261 (mouse glioblastoma cell line) and U251MG (human glioblastoma cell line) treated 6 h (A, E), 24 h (B, F), 48 h (C, G), 72 h (D, H) with temozolomide, astaxanthin or adonixanthin**.**

Data are shown as mean ± SEM (n = 6). **p* < 0.05, ***p* < 0.01 vs. control group (Tukey's test). TMZ; temozolomide.

A

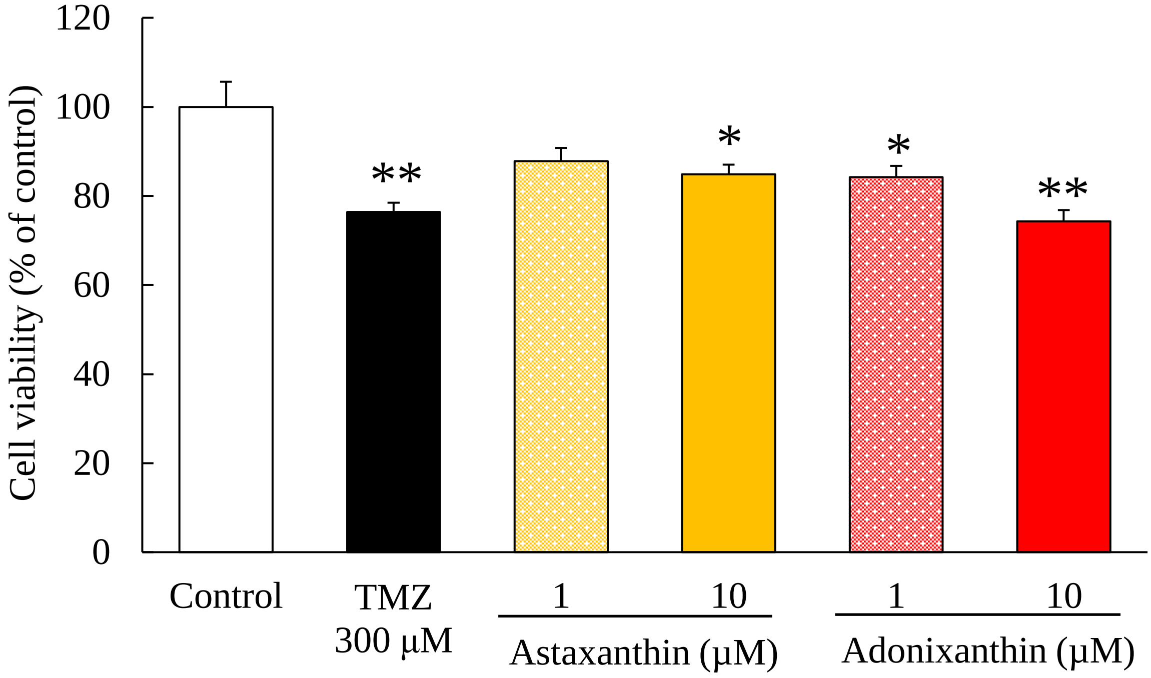

Supplemental figure 2. Cell viability of human glioblastoma cell line U87MG with astaxanthin and adonixanthin. This graph show the cell viability of human glioblastoma cell line U87MG treated 96 h with temozolomide, astaxanthin or adonixanthin. Data are shown as mean ± SEM (n = 6). *$p < 0.05$, **$p < 0.01$ vs. control group (Tukey's test). TMZ; temozolomide.

Supplemental Figure 3

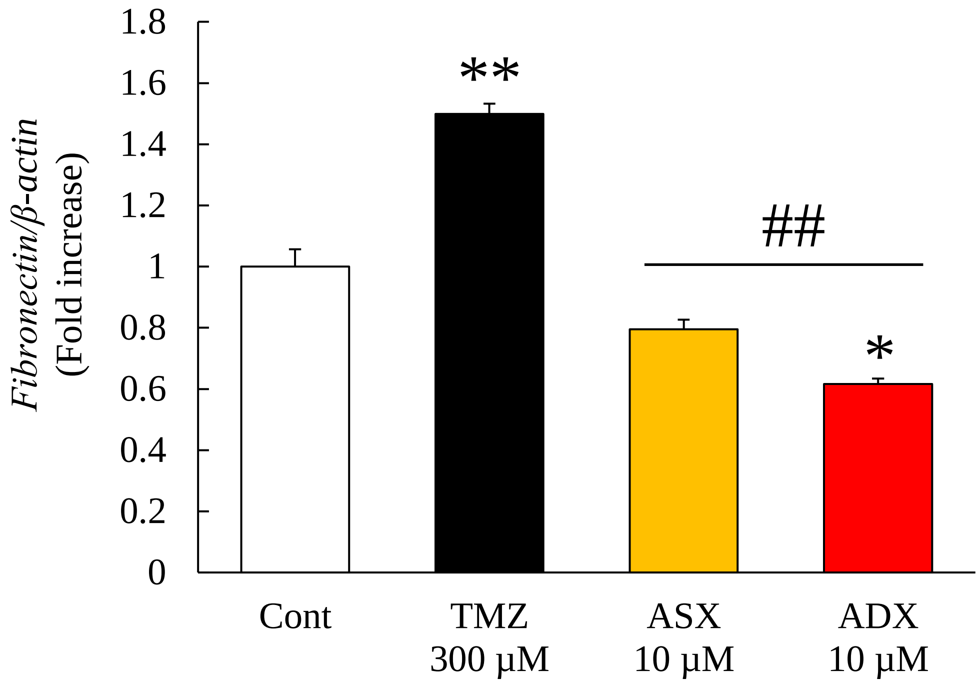

Supplemental figure 3. Expression of Fibronectin mRNA after 48h treatment of astaxanthin and adonixanthin in mouse glioblastoma cell line.

The quantitative data of fibronectin mRNA level in mouse glioblastoma cell line GL261 at 48 h after treatment of 300 μM temozolomide, 10 μM astaxanthin or 10 μM adonixanthin. Data are shown as mean ± SEM (n = 6). *$p < 0.05$, **$p < 0.01$ vs. control group (Student's *t*-test), ##$p < 0.01$ vs. astaxanthin group (Student's *t*-test) , TMZ; temozolomide. ASX; astaxanthin. ADX; adonixanthin.

Supplemental Figure 4

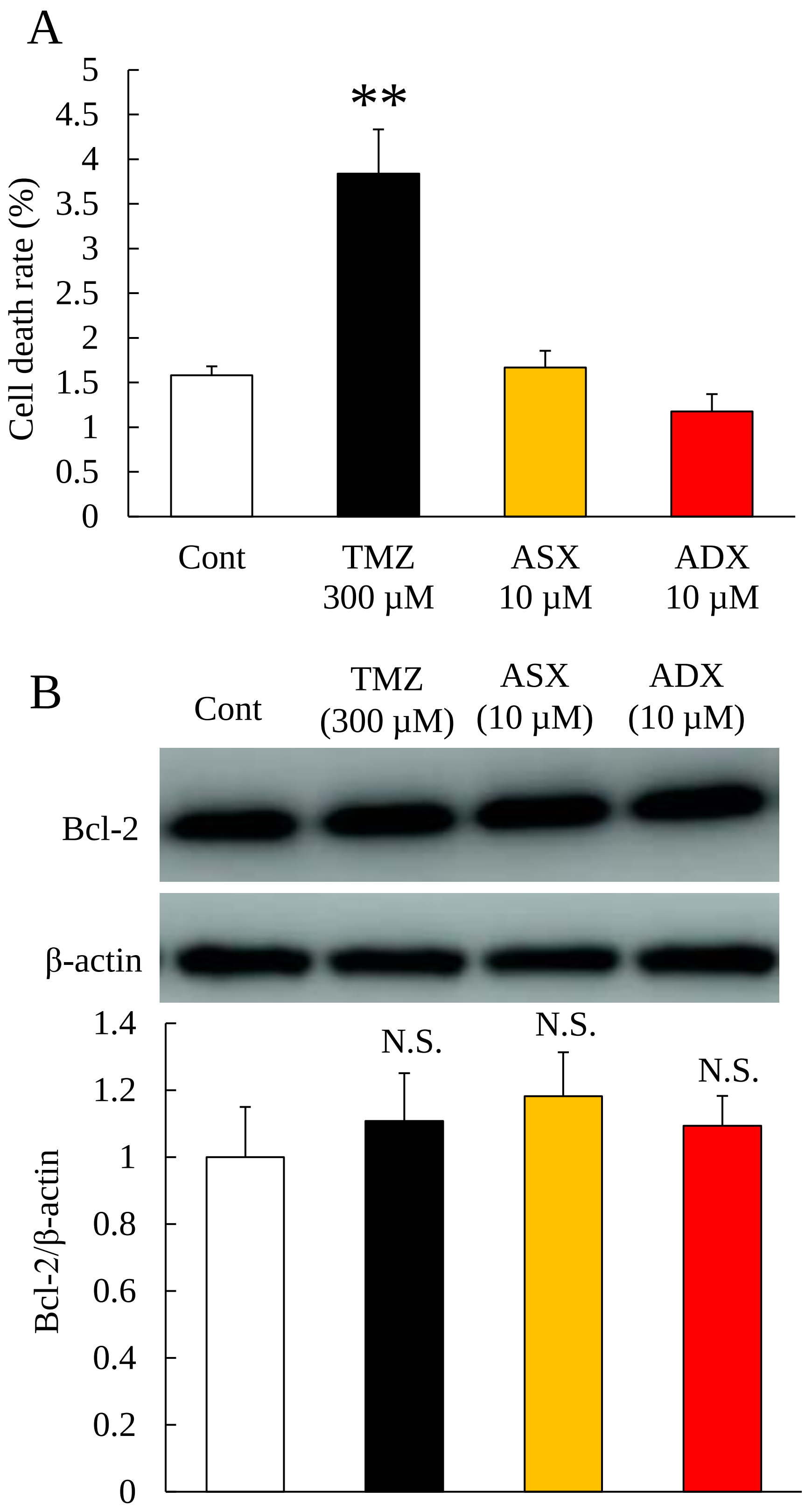

Supplemental figure 4 . The effect of astaxanthin and adonixanthin for cell death

(A) Cell death assay after 72h treatment of astaxanthin, adonixanthin and temozolomide. Data are shown as mean ± SEM (n = 6). $^{**}p < 0.01$ vs. control group (Student's *t*-test).

(B) Expression of Bcl-2 after 48h treatment of astaxanthin and adonixanthin in mouse glioblastoma cell line. Data are shown as mean ± SEM (n = 4). N.S. ; not significant (Student's *t*-test). TMZ; temozolomide. ASX; astaxanthin. ADX; adonixanthin.

Supplemental Figure 5

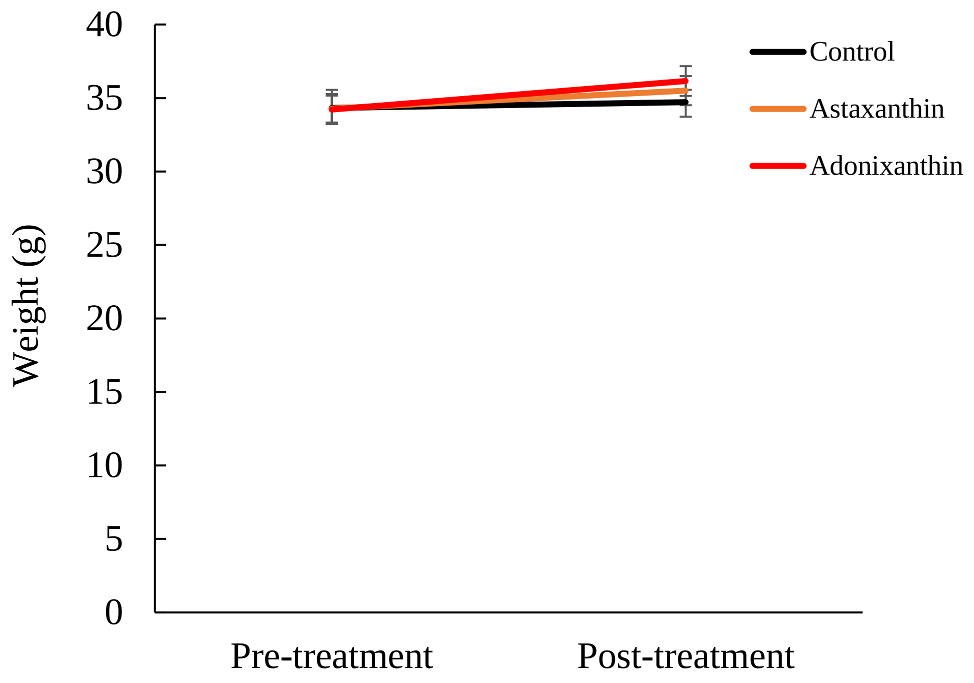

Supplemental figure 5. The effects of astaxanthin and adonixanthin on body weight.

This graph show the weight of mice at pre- or post-treated with astaxanthin or adonixanthin.

Data are shown as mean ± SEM (n = 4).
